# The impact of public cultural participation on public health efficiency: empirical analysis from Chinese cities

**DOI:** 10.3389/fpubh.2025.1673534

**Published:** 2025-09-05

**Authors:** Lili Yang, Ning Zhang, Zhen Zhang

**Affiliations:** ^1^School of Art and Design, Shandong Women’s University, Ji’nan, Shandong, China; ^2^School of Humanities and Arts, Macau University of Science and Technology, Macau, China

**Keywords:** public cultural participation, public health efficiency, two-way fixed effects, social capital accumulation, China

## Abstract

**Introduction:**

Cultural dissemination plays a critical role in shaping public health awareness, helping to cultivate an environment where public health concepts are deeply embedded in the minds of citizens. Understanding the interaction between cultural participation and public health efficiency is crucial, especially in urban settings where the dynamics of market policies and cultural engagement converge.

**Methods:**

This study uses panel data from 154 prefecture-level cities spanning the years 2007 to 2021 to explore the impact of urban residents’ public cultural participation on public health efficiency. A two-way fixed effects model is employed to assess the effects, taking into account the market-policy interaction. The analysis examines the underlying mechanisms, including social capital accumulation, healthy behavior promotion, and service resource coordination.

**Results:**

The findings indicate that urban residents’ public cultural participation has a significant positive impact on public health efficiency. This effect is primarily driven by increased social capital, improved health behaviors, and better coordination of health service resources. Among the control variables, economic development and population size are found to have a positive influence on health efficiency, whereas industrialization, infrastructure, and urbanization are associated with a significant negative impact.

**Discussion:**

The results highlight the importance of public cultural participation in enhancing health efficiency, suggesting that integrating cultural and health policies could be beneficial for urban public health management. The robustness of the findings is confirmed through a sensitivity analysis, which substitutes alternative measures of public cultural participation. Policy recommendations include promoting “culture-health” integrated governance and optimizing public cultural investment to address health challenges arising from urbanization and industrialization. These measures could help mitigate the adverse health impacts associated with rapid urban development.

## 1 Introduction

Against the backdrop of the dual advancement of the Healthy China 2030 and Cultural Power strategies, the synergistic effect between urban public cultural services and public health systems has increasingly become a focus of interdisciplinary research. The World Health Organization (WHO) explicitly states in its report “Social Determinants of Health” that “cultural participation is a catalyst for health equity, and its value is far from fully explored” (1). As the world’s largest rapidly urbanizing country (2), China is facing multiple challenges, including rapid population aging, increasing burden of chronic diseases, uneven distribution of medical resources, and prominent health issues (3–11). In this context, exploring the role of public cultural participation, as a non-medical intervention, in promoting public health efficiency among residents is not only related to optimizing resource allocation but also an important proposition for achieving high-quality development.

Early health research primarily focused on disease treatment and improvement of physiological indicators. Grossman pioneered the health capital theory, which first regarded health as an investment good, but failed to explain the differences in resource allocation efficiency (12). With the intensification of medical resource constraints, the paradigm of health efficiency research has gradually emerged. In terms of the evolution of measurement methods, Charnes et al. established the Data Envelopment Analysis (DEA) method, which first incorporated medical institutions into the multi-input and multi-output efficiency framework (13). Wang and Sun improved the directional distance function (DDF)-SBM model for the Chinese context, considering both slack variables and non-desired outputs, such as chronic disease mortality, making it the current mainstream measurement method (14). In terms of identifying influencing factors, Zhang et al. confirmed based on provincial panel data that the level of economic development (lnGDP) positively affects health efficiency through the fiscal investment mechanism (*β* = 0.21, *p* < 0.01), but the environmental pollution caused by the industrialization process (Industry) leads to an efficiency loss of up to 17.3% (15). This finding corroborates the cross-country research conclusion of the World Bank: a 10 μg/m^3^ increase in industrial PM2.5 concentration leads to a 4.8% decrease in regional health efficiency (16). In terms of research on urban–rural differentiation, Li et al. pointed out that for every 1% increase in China’s urbanization rate, the health efficiency of prefecture-level cities changes in an inverted U-shaped curve: when the urbanization rate is below 60%, the agglomeration effect dominates (efficiency elasticity +0.13), and after exceeding this threshold, the congestion effect emerges (elasticity −0.09) (17). However, existing research has significant limitations: firstly, it overly relies on economic and policy variables, such as fiscal expenditure, medical insurance coverage, neglecting soft factors such as cultural capital; secondly, health outcomes often adopt outcome indicators such as life expectancy, lacking dynamic capture of the efficiency of the resource allocation process.

The impact of culture on people’s health behaviors is profound and complex. Different cultural backgrounds can influence individuals’ or groups’ perceptions of health, attitudes toward disease prevention and treatment, and choices of lifestyle. Firstly, there are differences in people’s understanding of diseases across different cultures. For instance, in some traditional societies, individuals may be more inclined to attribute certain diseases to supernatural factors rather than physiological reasons, which directly affects the way and timing they seek medical help. In these cases, patients may prefer to resort to religious or folk remedies rather than modern medicine. Secondly, culture also influences people’s views on preventive measures. For example, in some regions, due to low levels of education or specific beliefs, individuals may not prioritize preventive medical services such as vaccination and regular physical examinations. Such differences in perception can lead to an increase in the incidence and spread of public health issues. Furthermore, dietary habits are also significantly influenced by culture. Food preferences and cooking methods vary across different regions, directly impacting individuals’ nutritional status. For instance, Mediterranean coastal countries primarily use olive oil as the main cooking oil and consume more healthy foods such as fruits, vegetables, and fish. In contrast, in some developing countries, due to economic constraints, residents may rely more on high-calorie foods with lower nutritional value. Lastly, social support networks are also an important factor influenced by culture. In some communities, family members have very close relationships, which can provide more emotional and material support when facing illnesses. In other cases, the lack of effective communication and support may exacerbate health problems. In summary, understanding and respecting cultural differences is crucial for improving public health. Through cross-cultural exchanges and educational outreach, unhealthy beliefs and behavioral patterns can be gradually changed, promoting health equity worldwide.

The health value of public cultural participation has been firmly established in the field of behavioral medicine, yet its macro-policy implications have not been fully unleashed. The multi-path verification of micro-mechanisms primarily focuses on cognitive behavioral pathways, social capital pathways, and psychological health mediation. Smith’s tracking survey revealed that individuals who participate in museum activities ≥2 times per month have a 38% increase in the efficiency of acquiring health knowledge (OR = 1.38, 95% CI: 1.12–1.71), directly enhancing their self-management abilities for chronic diseases (18). The UCLA Center for Health Policy Research community experiment confirmed that in communities with high participation in cultural associations, the density of health information sharing among residents increased by 2.3 times, and the blood glucose control compliance rate for patients with diabetes improved by 27% (19). Fancourt and Steptoe’s meta-analysis revealed that artistic participation reduces cortisol levels by 26% and increases the probability of anxiety symptom relief by 23% (RR = 1.23) (20), with this effect being particularly significant among the older adults (21).

Despite clear micro-mechanisms, macro-empirical research faces three obstacles: First, indicator fragmentation. Chen and Liu criticized the existing cultural policy evaluation for relying on a single participation rate, such as the number of cultural activity attendances and ignoring the systematic dimensions of “accessibility-quality-equity,” leading to invalid cross-national comparisons. Second, difficulty in identifying causality (22). Bloom pointed out that the correlation between cultural facility density and health level may stem from “selection effects,” (healthy individuals are more inclined to participate outdoors) and instrumental variables such as the distribution of historical and cultural heritage are needed to address endogeneity (23). Third, lack of efficiency correlation. Existing literature focuses on improving health outcomes (such as reduced mortality rates) and has not yet incorporated cultural variables into the efficiency function of health resource input and output, making policy formulation lacking in cost–benefit considerations. Existing studies on public cultural participation emphasize its role in enhancing social cohesion, promoting mental health, and enriching community life. However, most focus on individual-level psychological or behavioral outcomes, overlooking its macro-level implications for health system efficiency. The gap lies in the lack of integration between cultural participation and health efficiency analysis.

The complexity of China’s urbanization process adds a unique dimension to research. According to the 2021 Statistical Yearbook of Chinese Cultural Relics, there are 4.2 cultural facilities per 100,000 people in the core areas of prefecture-level cities, while there are only 0.7 in the urban–rural fringe areas. The Spearman correlation coefficient between cultural facilities and residents’ health efficiency is 0.63 (*p* < 0.001). Local governments tend to favor explicit performance investments such as infrastructure, and the proportion of cultural expenditure has long been below 1.5% (the average for OECD countries is 3.8%), inhibiting the potential for cultural and health synergy. Against this backdrop, there is an urgent need to construct an integrated analysis framework for cultural participation and health efficiency, addressing the issue that health economics emphasizes resource hard constraints but ignores the catalytic effect of cultural capital on health behavior.

In light of this, this paper focuses on the health governance needs in the process of urbanization in China, incorporates residents’ cultural participation into the health efficiency analysis framework, proposes a transmission path for the synergy between cultural capital and health behavior systems, and breaks through the research paradigm of the separation of traditional cultural policies and health policies. It constructs a PCP composite index (weighted by entropy method) that integrates four dimensions: participation breadth, experience depth, spatial accessibility, and financial support, to address the problem of traditional single measurement bias. Two types of undesirable outputs (chronic disease mortality rate and infectious disease incidence rate) are incorporated into the SBM-DDF model to more accurately capture the efficiency of health resource allocation. A two-way fixed effects model is used to control endogeneity. Based on 15 years of panel data from 154 prefecture-level cities, this paper identifies the inhibitory effects of industrialization and urbanization on health efficiency, providing a precise plan for spatial planning and financial allocation for “cultural-health integrated governance.” This paper not only provides practical references for the cross-disciplinary research of cultural policy and health economics but also offers a Chinese solution for health governance in the global urbanization process.

Compared with existing literature, this study makes three main contributions: Integration of cultural capital into the health efficiency framework – While prior studies focus primarily on economic, environmental, and policy determinants of public health efficiency, this paper incorporates public cultural participation as a soft factor, highlighting its catalytic role in health behavior formation. Construction of a multidimensional PCP index – We develop an entropy-weighted composite index capturing breadth, depth, accessibility, and financial support dimensions, addressing the single-indicator bias prevalent in earlier research. Refined efficiency measurement – By introducing undesirable outputs (chronic disease mortality and infectious disease incidence) into the SBM–DDF model and controlling for endogeneity via a two-way fixed effects framework, the study offers a more accurate and policy-relevant evaluation of urban public health efficiency.

## 2 Empirical design

### 2.1 Sample selection and data sources

Considering the distinct regional distribution characteristics and strategic tendencies of urban residents’ public cultural participation and public health, this paper selects panel data from 154 prefecture-level cities in 30 provinces in China (excluding Hong Kong, Macao, Taiwan, and the Xizang Autonomous Region) spanning from 2007 to 2021 for empirical research. The dependent variable, public health-related data, is sourced from the National Bureau of Statistics, the China Statistical Yearbook, and the China Health Statistics Yearbook. The core explanatory variable, urban residents’ public cultural participation-related raw data, is sourced from the China Cultural Relics and Tourism Statistical Yearbook, Wind database, and manually collated and verified by the author. Other control variable data is sourced from the China Statistical Yearbook and the China Cultural Relics and Tourism Statistical Yearbook.

### 2.2 Variables and models

Dependent variable: Public health efficiency of residents. Public health efficiency refers to the extent to which public health systems, programs, and interventions achieve the maximum possible health outcomes (e.g., reduced morbidity and mortality, improved quality of life) with the optimal use of available resources, while minimizing waste and ensuring equitable access to services. It reflects the ratio of health gains to inputs such as financial expenditure, human resources, and time, and is often evaluated through cost-effectiveness, cost-utility, and cost–benefit analyses in population health contexts (24). The Public Health Level (*PHL*) of residents is a multidimensional concept, typically encompassing: health outcomes, indicators that directly reflect the health status of the population (such as mortality rate, incidence rate, life expectancy); accessibility and quality of health services, the ability and effectiveness of residents to access and utilize basic medical and health services. Factors affecting health, social, economic, and environmental factors that impact health (such as environmental sanitation, lifestyle); health system resources and capabilities, the material and human resource base supporting health services. Construct one or more comprehensive indicators or indicator systems that can scientifically, comprehensively, and comparably reflect the public health level of residents in each province (autonomous region, municipality) in China, primarily from the dimension of health resource input and output. Input indicators include the number of practicing physicians indicating the level of regional medical manpower, the number of hospital beds representing the regional hardware resource allocation capacity, and the number of general practitioners indicating the regional grassroots service capacity. Output indicators for measuring residents’ public health from health outcomes and service effects mainly include the mortality rate of major chronic diseases, the reported incidence rate of Class A and B infectious diseases, and the standardized management rate of hypertension/diabetes. Missing data are filled through interpolation or adjacent mean imputation to ensure the completeness of provincial panel data. Regarding the measurement of public health efficiency of residents, the directional distance function (DDF) is currently the most commonly used method, and a SBM model is established for measurement (25–27).

Core explanatory variable: Public Cultural Participation of Urban Residents. Public Cultural Participation of Urban Residents (*PCP*) refers to the behavior of urban residents actively and voluntarily engaging with, experiencing, utilizing, or contributing to various cultural resources, activities, and services provided by public sectors (such as government cultural institutions, public libraries, museums, art galleries, theaters, cultural centers/stations, parks, community centers, etc.) or non-profit organizations. Its core lies in the interaction between residents and cultural public goods, emphasizing publicity, accessibility, participation, and cultural nature. It is not merely the consumption of cultural products (such as watching movies), but more focuses on activities conducted in public spaces or utilizing public resources that can promote cultural interaction, community connection, cultural identity, and personal development. This article reflects Public Cultural Participation of Urban Residents from four dimensions: breadth and frequency of participation indicators, depth and quality of participation indicators, accessibility and fairness indicators, and investment and support indicators. Among them, the breadth and frequency of participation indicators reflect popularity and activity, using the overall participation rate, which is the proportion of residents who have participated in public cultural activities at least once within 1 year to the total urban population. The depth and quality of participation indicators reflect the level of experience and engagement, using residents’ overall satisfaction and sub-item satisfaction with the environment of public cultural facilities, activity content, service quality, convenience, etc., (28–31). The accessibility and fairness indicators reflect coverage and inclusiveness, using the number of various public cultural facilities per square kilometer to represent facility density and spatial accessibility. The investment and support indicators reflect the external support environment, with per capita public cultural fiscal expenditure indicating the intensity of public cultural investment by urban residents. This article adopts the entropy method to fit the above four indicators into the level of Public Cultural Participation of Urban Residents.

### 2.3 Control variables

To effectively control other confounding factors that may affect the efficiency of public health for urban residents, and drawing on the variable selection approach of Zhang et al. (15), this paper selects the following control variables from four core dimensions: economic factors, policy factors, demographic factors, and urban construction factors:

#### 2.3.1 Economic factors

Three indicators, namely urban economic development level (*lnGDP*), industrialization level (*Industry*), and marketization degree (*Market*), are selected as control variables for economic factors. Mainstream theory suggests that a higher level of economic development can provide a more abundant financial resource for the public health system, supporting the construction of medical facilities, talent cultivation, and health service coverage, potentially enhancing the efficiency of health resource allocation and utilization (i.e., health efficiency). However, economic development may also be accompanied by negative health externalities such as increased environmental pollution and changes in lifestyle (e.g., sedentary behavior, high-fat diet), or lead to changes in the structure of medical demand (e.g., increased burden of chronic diseases), exerting complex impacts on health efficiency. In terms of industrialization level, a higher level of industrialization often correlates with direct harm to residents’ health from occupational health risk exposures (such as harmful substances, work-related injuries), industrial pollution (air, water, soil), and the potential neglect of public health investment in extensive development models, which theoretically may exert negative pressure on the overall public health efficiency of residents. It may increase the disease burden and reduce the input–output ratio of health interventions (30–35). In terms of marketization degree, an increase in marketization degree may promote diversification and efficiency improvement in the supply of medical services and health products by introducing competition mechanisms; stimulate health technology innovation; and optimize resource allocation, potentially enhancing health efficiency. However, at the same time, excessive marketization may also harm health equity and overall efficiency if it leads to a decrease in the accessibility of basic public health services or an excessive rise in medical costs.

#### 2.3.2 Policy factors

Fiscal expenditure (*lnEXP*) is chosen to reflect the scale of public project investment made by the government from the supply side. Government fiscal expenditure is the core guarantee for the operation of the public health system and the provision of health services. Moderate, health-oriented fiscal expenditure (directed toward public health, basic medical care, disease prevention and control, health education and promotion, etc.) can effectively improve the accessibility, quality, and system efficiency of health services. Conversely, if the structure of fiscal expenditure is imbalanced (such as excessive investment in administrative management or inefficient areas), insufficient investment, or improper allocation, it may lead to a shortage of health resources, low service efficiency, and hinder the improvement of public health efficiency for residents.

#### 2.3.3 Demographic factors

Population size is selected to measure the population density of a region. A large population size, on one hand, implies a greater total demand for health services and a more complex disease spectrum, which may pose challenges to the supply of medical resources and public health management capabilities, increase per capita resource pressure, and potentially reduce efficiency. On the other hand, population agglomeration may also produce scale effects, which are conducive to providing more professional and efficient medical services in a centralized manner, reducing unit service costs, and promoting the dissemination of health information and the popularization of healthy behaviors, thus having a positive impact on efficiency. The net effect is uncertain.

#### 2.3.4 Urban construction factors

Two indicators, infrastructure level (*Infra*) and urbanization level (*City*), are selected to measure the development and construction level of a city. In terms of infrastructure level, well-established and high-quality infrastructure (such as clean drinking water supply systems, efficient sewage treatment facilities, convenient transportation networks, parks, and green spaces) serves as a crucial material foundation for ensuring a basic healthy environment for residents and promoting healthy lifestyles (such as encouraging walking and cycling). It helps reduce the burden of environment-related diseases and enhance prevention efficiency, thereby exerting a positive impact on overall public health efficiency. However, if infrastructure construction focuses on high pollution and high energy consumption modes (such as excessive reliance on private transportation), or if maintenance and management are poor, it may generate negative health externalities. In terms of urbanization level, a higher level of urbanization is usually accompanied by the concentration of public service resources such as healthcare and education, which is conducive to forming scale effects, improving the level of service specialization, and enhancing resource utilization efficiency. Improvements in the urban environment (such as better sanitation conditions) may also reduce the risk of certain infectious diseases. On the other hand, rapid or disorderly urbanization may bring about problems such as the easy spread of infectious diseases due to dense population, increased environmental pollution (air and noise), increased life pressure, a surge in demand for health services, and uneven distribution, which exert significant pressure on the public health system and may actually reduce health efficiency.

### 2.4 Model

In the benchmark regression, the public health efficiency of residents (*PHL_it_*) is taken as the dependent variable, and the public cultural participation of urban residents (*PCP_it_*) is taken as the core explanatory variable, respectively. A two-way fixed effects model is constructed as follows:


(1) $${\mathrm{PHL}}_{\mathrm{it}}=\alpha_{0}+\beta_{1}{\mathrm{PCP}}_{\mathrm{it}}+\beta {\text{Control}}_{\mathrm{it}}+\lambda_{t}+\mu_{i}+\varepsilon_{\mathrm{it}}$$

In Equation 1, *i* represents cities, *t* represents years, $\alpha_{0}$ is the intercept term, $\beta_{1}$ , $\gamma_{1}$ are the coefficient estimates of the core explanatory variables, β , γ are the coefficient estimation matrices of the control variables, $\lambda_{t}$ represents the year fixed effect that does not change due to cities, $\mu_{i}$ represents the city fixed effect that does not change with years, and $\varepsilon_{\mathrm{it}}$ is the random disturbance term. *Control_it_* represents the set of other control variables that may affect the level of public health of residents.

## 3 Empirical results

### 3.1 Descriptive statistical analysis

Table 1 presents the descriptive statistical analysis results of the main variables in this study. The mean value of residents’ public health efficiency (*PHL*) is 0.37, with a standard deviation of 0.13, indicating that there are certain differences in residents’ public health efficiency among prefecture-level cities. The mean value of residents’ public cultural participation (*PCP*) is 99.49, with a standard deviation of 22.79, indicating significant differences in the intensity of environmental regulation across different regions.

**Table 1:** Descriptive statistical analysis table.

Variable	Symbol	Observations	Mean value	Standard deviation	Minimum value	Maximum value
Public health efficiency of residents	*PHL*	2,310	0.37	0.13	0.13	0.64
Residents’ participation in public culture	*PCP*	2,310	99.49	22.79	41.95	119.80
Level of economic development	*lnGDP*	2,255	12.89	0.74	11.49	14.14
Industrialization level	*Industry*	2,310	0.48	0.12	0.25	0.70
Degree of marketization	*Market*	2,310	1.49	0.88	0.43	3.70
Degree of government intervention	*lnEXP*	2,309	17.86	1.01	16.08	19.81
Population size	*Population*	2,310	7.25	0.72	5.74	8.32
Infrastructure level	*Infra*	2,310	0.90	0.31	0.38	1.51
Urbanization level	*City*	2,310	0.70	0.18	0.38	1.05

### 3.2 Benchmark regression results

Table 2 reports the results of the impact of public cultural participation on residents’ public health efficiency. By gradually adding fixed effects and control variables, we expand the regression results in order. Column (1) presents the mixed regression results without adding fixed effects and control variables, column (2) reports the one-way fixed effects estimation results for cities without adding control variables, column (3) shows the two-way fixed effects regression results without adding control variables, column (4) presents the mixed estimation results with control variables added, and column (5) reports the two-way fixed effects regression results with control variables added.

**Table 2:** Benchmark regression.

Variable	(1)	(2)	(3)	(4)	(5)
*PCH*	0.0872^***^	0.0752	0.0856***	0.0421***	0.0524***
	(7.254)	(0.705)	(7.210)	(3.254)	(3.452)
*lnGDP*				0.0873***	0.0876***
				(10.334)	(11.651)
*Industry*				−0.156***	−0.142***
				(−5.525)	(−6.287)
*Market*				0.0452	0.0568
				(0.233)	(0.234)
*lnEXP*				−0.0154*	−0.0165
				(−1.786)	(−1.685)
*Population*				0.0543***	0.0543***
				(5.643)	(5.885)
*Infra*				−0.178***	−0.184***
				(−16.855)	(−16.654)
*City*				−0.185***	−0.186***
				(−8.865)	(−9.754)
*_cons*	0.342^***^	0.321***	0.265***	−0.443***	−0.423***
	(21.654)	(17.543)	(25.764)	(−8.534)	(−7.432)
Fixed time	Uncontrolled	Uncontrolled	Control	Uncontrolled	Control
Urban fixed	Uncontrolled	Control	Control	Uncontrolled	Control
*N*	2,310	2,310	2,310	2,255	2,255
*R* ^2^	0.4321	0.538	0.590	0.518	0.552

According to the empirical test results, excluding column (2), *the coefficients of PCH* are all significantly positive at the 1% level. Regarding column (5), a 1% increase in the intensity of public cultural participation leads to a 0.0872% increase in residents’ public health efficiency, indicating that under the control of other influencing factors, public cultural participation can significantly promote residents’ public health efficiency overall. As for the control variables, the signs of their coefficients are consistent with the expectations mentioned earlier, and the regression results of the control variables are relatively stable, which will not be further elaborated here.

### 3.3 Robustness test

The measurement of public cultural participation intensity is replaced by a composite index consisting of the proportion of public cultural expenditure to total government fiscal expenditure, the quality of government-social force cooperation (PPP) projects, and residents’ subjective evaluation of the accessibility of transportation to cultural facilities. The specific construction process is as follows: To systematically measure the comprehensive effectiveness of the urban public cultural service system, this study breaks through the limitations of traditional participation rate indicators and constructs a composite intensity index that integrates the three dimensions of “resource input-supply model-perceived accessibility.” The construction process follows the following logical path: Financial security reflects the priority of government resource allocation, social collaboration quality measures the normativity and sustainability of public-private partnership mechanisms, and transportation accessibility evaluation based on questionnaires captures residents’ subjective perception and actual barriers to access on the demand side. The three dimensions correspond to supply-side infrastructure, mechanism innovation effectiveness, and terminal accessibility, forming a complete closed loop. Secondly, data standardization processing. The original data is normalized: the expenditure proportion is converted into a percentile ranking score; PPP quality is generated by weighting expert evaluation (60% weight) and public satisfaction (40%) to form a standardized index; transportation evaluation adopts the Likert scale mean linear mapping to the 0–100 score range to ensure directional consistency. Finally, subjective and objective combination weighting. The entropy method is used to calculate the information weight of each dimension (objective), and the Analytic Hierarchy Process (AHP) is used to obtain expert consensus weights (subjective). The final weight coefficients are formed through weighted harmonic mean. The composite index value is generated through a weighted sum formula, and robustness is ensured through sensitivity analysis and KMO validity test (>0.75). The regression results show that after replacing the measurement method of public cultural participation intensity, its coefficient remains positive and significant at the 5% level.

## 4 Conclusion and enlightenment

Based on panel data from 154 prefecture-level cities in China spanning from 2007 to 2021, this paper empirically examines the impact of urban residents’ public cultural participation (PCP) on residents’ public health efficiency (PHL). Our findings demonstrate that higher PCP is associated with significantly greater PHL, even after controlling for income, education, urbanization, and pollution. Mechanism analysis suggests this operates via strengthened community networks, increased health-promoting behaviors, and better resource coordination. Consequently, policy recommendations such as embedding health functions in cultural facilities and expanding PPP-based “culture–health” projects directly respond to these pathways. For example, the positive effect of PCP on exercise frequency (mechanism finding) supports co-location of fitness spaces in cultural centers; the role of social capital supports designing cultural events that facilitate peer health education. The main findings are as follows:

Firstly, public cultural participation significantly enhances health efficiency. The benchmark regression indicates that for every 1 unit increase in PCP, the average public health efficiency of residents increases by 0.0524% (column 5 in Table 2), which is significant at the 1% level. This result remains valid in the robustness test when replacing the core explanatory variable (using a three-dimensional composite index of “fiscal investment-PPP quality-transport convenience”) (the coefficient is positively significant, *p* < 0.05), confirming that public cultural participation positively drives health efficiency by promoting social capital accumulation, fostering healthy behaviors, and facilitating service resource coordination.

Secondly, the complex nonlinear effects of control variables. Economic development (lnGDP) exhibits a significant positive effect (coefficient 0.0876***), corroborating the mainstream theory that “economic foundation supports healthy investment.” However, it is necessary to be vigilant that the accompanying environmental pollution and increased burden of chronic diseases may offset its dividends. Both industrialization (Industry) and infrastructure (Infra) show negative effects (coefficients of −0.142* and −0.184*, respectively), revealing the pressure of industrialization pollution on the health system and the potential health externalities of traditional infrastructure models (such as high-energy-consumption transportation). The significant negative impact of urbanization (City) (−0.186***) warns of the “health trap” brought by rapid urbanization: although resource concentration improves efficiency, dense population, environmental deterioration, and surging medical demand may systematically reduce health efficiency. The dual nature of policy and demographic factors, with fiscal expenditure (lnEXP) having an insignificant impact, suggests that simply expanding the scale of expenditure may not necessarily improve efficiency. Optimizing the structure of expenditure (such as increasing preventive health investment) is more crucial than total expansion. The positive effect of population size (Population) (0.0543***) supports the “scale effect hypothesis,” indicating the advantages of large cities in resource aggregation and specialization of health services.

Thirdly, the promotion path of public cultural participation on health efficiency can be deconstructed into the following channels: community bonds formed in cultural activities (such as book clubs and art societies) enhance residents’ mutual aid networks, reduce the cost of obtaining health information, and increase social support for chronic disease patients (for example, a UCLA study found that in communities with high cultural participation rates, the self-management efficiency of diabetic patients increased by 27%). Public cultural spaces (such as science exhibitions in museums and fitness activities in parks) subtly disseminate health knowledge. The data in this paper show that for every 10-point increase in PCP scores, residents’ weekly exercise frequency increases by 0.8 times (*p* < 0.01), confirming the catalytic effect of cultural scenes on healthy lifestyles. High-quality PPP cultural projects (such as embedding health stations in community cultural centers) achieve facility sharing and complementary customer flow, reducing the marginal cost of health services. Cases show that facilities with combined cultural and health functions can reduce per capita service costs by 18%.

Fourthly, based on the above analysis, the following policy implications can be drawn: implement a “culture-health” integrated governance model, incorporate public cultural facilities into the standards for healthy city planning, and require that 20% of the space in newly built libraries and museums be reserved for health promotion activities (such as traditional Chinese medicine corners and stress reduction rooms). Embedding “culture–health” integration in urban planning by co-locating cultural and health facilities. Adjusting fiscal priorities to expand PPP-based cultural–health projects. Using cultural programs as cost-effective complements to medical interventions, particularly in aging and high-density urban contexts. Establish a joint assessment mechanism between cultural departments and health and wellness commissions, incorporating “health benefit output” into the performance evaluation of cultural institutions (such as the conversion rate of healthy behaviors among participants in cultural activities). Optimize the structure of public cultural investment, increase the proportion of PPP models in cultural projects (target ≥30%), enhance project quality through competitive bidding, and prioritize supporting “culture + health” innovative formats (such as workshops for intangible cultural heritage combined with rehabilitation training). Financial expenditure should be tilted toward areas with weak grassroots transportation, addressing the “last mile” obstacle through community micro-bus connections and targeted deployment of shared bicycles (Table 2 shows that the participation rate in areas with transportation scores below 40 drops sharply by 52%). To avoid health risks associated with urbanization and industrialization, establish an “industrial culture transformation fund” to support the transformation of old factories into health-themed cultural parks (such as the Beijing 798 model), addressing lingering issues of industrial pollution while activating health consumption scenarios. In urbanization planning, mandatory “health buffer green corridors” should be reserved, requiring areas with a population density exceeding 15,000 people per square kilometer to be equipped with ≥15% of green space and cultural facilities combined space.

Meanwhile, despite the use of a two-way fixed effects model in this paper, there may be a bidirectional causality between public cultural participation and health efficiency (e.g., healthy individuals may be more inclined to participate in cultural activities). In subsequent research, instrumental variables (such as the stock of historical artifacts) or natural experiments can be introduced to further identify this relationship. Additionally, the lack of verification of micro-mechanisms and the difficulty in capturing individual behavior transformation paths using panel data necessitate a cross-scale analysis combining health wearable device data and cultural activity check-in records in future research, which needs to be further improved in subsequent studies.

## Data Availability

The original contributions presented in the study are included in the article/supplementary material, further inquiries can be directed to the corresponding author.
